# Identification and bioinformatics analysis of genes associated with pyroptosis in spinal cord injury of rat and mouse

**DOI:** 10.1038/s41598-024-64843-6

**Published:** 2024-06-18

**Authors:** Fu-Sheng Liu, Hai-Long Huang, Lin-Xia Deng, Qian-Shi Zhang, Xiao-Bin Wang, Jing Li, Fu-Bing Liu

**Affiliations:** 1 Department of Spine Surgery, The Second Xiangya Hospital, Central South University, 139 Renmin Middle Road, Changsha, 410011 Hunan China; 2 Department of Neurosurgery, Xiangya Hospital, Central South University, Changsha, 410011 China; 3 Department of Pediatrics, The Third Xiangya Hospital, Central South University, Changsha, 410011 China

**Keywords:** Spinal cord injury, Bioinformatics analysis, Pyroptosis, Differential expression analysis, SCI model, Molecular biology, Neuroscience, Neurology

## Abstract

The mechanism of spinal cord injury (SCI) is highly complex, and an increasing number of studies have indicated the involvement of pyroptosis in the physiological and pathological processes of secondary SCI. However, there is limited bioinformatics research on pyroptosis-related genes (PRGs) in SCI. This study aims to identify and validate differentially expressed PRGs in the GEO database, perform bioinformatics analysis, and construct regulatory networks to explore potential regulatory mechanisms and therapeutic targets for SCI. We obtained high-throughput sequencing datasets of SCI in rats and mice from the GEO database. Differential analysis was conducted using the “limma” package in R to identify differentially expressed genes (DEGs). These genes were then intersected with previously reported PRGs, resulting in a set of PRGs in SCI. GO and KEGG enrichment analyses, as well as correlation analysis, were performed on the PRGs in both rat and mouse models of SCI. Additionally, a protein–protein interaction (PPI) network was constructed using the STRING website to examine the relationships between proteins. Hub genes were identified using Cytoscape software, and the intersection of the top 5 hub genes in rats and mice were selected for subsequent experimentally validated. Furthermore, a competing endogenous RNA (ceRNA) network was constructed to explore potential regulatory mechanisms. The gene expression profiles of GSE93249, GSE133093, GSE138637, GSE174549, GSE45376, GSE171441_3d and GSE171441_35d were selected in this study. We identified 10 and 12 PRGs in rats and mice datasets respectively. Six common DEGs were identified in the intersection of rats and mice PRGs. Enrichment analysis of these DEGs indicated that GO analysis was mainly focused on inflammation-related factors, while KEGG analysis showed that the most genes were enriched on the NOD-like receptor signaling pathway. We constructed a ceRNA regulatory network that consisted of five important PRGs, as well as 24 miRNAs and 34 lncRNAs. This network revealed potential regulatory mechanisms. Additionally, the three hub genes obtained from the intersection were validated in the rat model, showing high expression of PRGs in SCI. Pyroptosis is involved in secondary SCI and may play a significant role in its pathogenesis. The regulatory mechanisms associated with pyroptosis deserve further in-depth research.

## Introduction

Spinal cord injury (SCI) refers to the disruption of the integrity or continuity of the spinal cord caused by various etiologies, resulting in temporary or permanent loss of sensory, motor, and other functions below the level of injury segment^1–3^. Despite remarkable advances in medical technology, treatment options for SCI, including surgery, medication, and rehabilitation, as well as emerging therapies such as stem cell transplantation and biomaterial therapy, are still unable to achieve complete clinical cure^4–10^. The complications and sequelae of SCI cause significant physical and psychological impairments to patients and impose a substantial burden on families and society^1,11^. Recent epidemiological studies indicate that the incidence of traumatic SCI is approximately 10.5 per 100,000 individuals, with nearly 800,000 new cases occurring globally each year^6,11–13^. Therefore, the investigation of the mechanisms underlying SCI and the exploration of novel therapeutic strategies hold important practical significance.

The mechanism underlying SCI is highly complex and broadly can be categorized into two types: primary and secondary injury^11^. Primary injury results from the immediate impact of the injurious factor on the spinal cord, leading to damage of spinal cord tissue^13^. In contrast, secondary injury is a cascade of complex physiological and pathological changes that occur following the primary injury. These changes include edema, vascular rupture, electrolyte imbalances, oxidative stress, and various forms of cell death^11,13–15^. Secondary injury is modifiable and thus forms the basis for numerous intervention strategies that aim to improve clinical outcomes. Recent studies have demonstrated that pyroptosis, a newly identified type of programmed cell death, contributes to the secondary injury of the spinal cord^16–18^. Pyroptosis is distinct from both apoptotic and necroptosis and is characterized by the formation of cytoplasmic membrane pores, cell swelling, cell membrane rupture, massive release of inflammatory factors, and cell death^18^. Pyroptosis is involved in various physiological and pathological processes and plays a regulatory role in the development and progression of a wide range of diseases, including infections, neurodegenerative disorders, brain injury, ischemia–reperfusion injury, atherosclerosis, metabolic disorders, and tumors, among others^19–22^. Although some research has explored the role of pyroptosis in SCI, there has been limited exploration of the specific molecular mechanisms and components of this form of programmed cell death in the context of SCI^23,24^. Further investigation in this area has the potential to improve our understanding of the pathogenesis of SCI and facilitate the development of novel treatment strategies.

The aim of this study is to collate and analyze the spinal cord tissue sequencing data from rat and mouse models of SCI in the GEO (gene expression omnubus) database (https://www.ncbi.nlm.nih.gov). The differentially expressed genes (DEGs) will be integrated with the pyroptosis-related genes (PRGs) to identify the PRGs network involved in the differential expression of SCI. This approach will enhance our understanding of the role of PRGs in SCI and help to elucidate the specific mechanisms of injury. Additionally, the identification of these genes may provide new targets for intervention in the treatment of SCI.

## Materials and methods

### Data source and data processing

We downloaded four high-throughput sequencing datasets of SCI in *Rattus norvegicus* (GSE93249, GSE133093, GSE138637 and GSE174549) and two high-throughput sequencing datasets in *Mus musculus* (GSE45376 and GSE171441) from the GEO database. In these datasets, we exclusively analyzed the sequencing results of the pure SCI group and the sham surgery group, excluding data from other treatment groups such as drug administration or electrical stimulation. Additionally, in the GSE171441 dataset, samples were collected for sequencing at 3 days and 35 days after SCI. We separated and compared these samples separately with the sham surgery group during data processing, and named the dataset as GSE171441_3d and GSE171441_35d respectively. PRGs data set was identified and synthesized from previous literature^18,25–29^ through a thorough review of full texts and corresponding references (Supplementary Table 1).

### Gene differential expression analysis

The “limma” R package was utilized to perform the differential expression analysis between SCI group and sham surgery group from GEO databases. |Log_2_(fold change)| ≥ 0.5 and *P* value < 0.05 were considered as the cutoff criteria. The volcano plot was drawn to show the DEGs.

### Protein–protein interaction (PPI) analysis

To explore the PPI network of the selected genes, they were imported into the STRING database (https://string-db.org), which is a web tool used to explore the interactions between multiple proteins.

### Functional enrichment analysis

Gene Ontology (GO) and Kyoto encyclopedia of genes and genomes^30,31^(KEGG, https://www.kegg.jp/kegg/kegg1.html) analyses were performed with the Database for Annotation, Visualization and Integrated Discovery (DAVID) to explore the mechanisms and pathways of DEGs.

### Animal groups and SCI model

All animal study protocols were approved by the Research Ethics Committee of The Second Xiangya Hospital, Central South University, Hunan, P.R. China, and all procedures were performed in accordance with ethical standards and ARRIVE guidelines. Female Sprague–Dawley (SD) rats, aged 10 weeks old, weighting between 200 and 230 g, were chosen as the experimental animal for this study, provided by the Department of laboratory Animals of Central South University. All rats were housed under a 12 h light/dark cycle pathogen-free condition with a controlled temperature of 23 ± 2 °C and 60 ± 5% humidity and free access to food and water. The rats were randomly divided into two groups: the sham surgery group and the SCI group, and 4 rats in each group.

The SCI model was established based on a modified Allen’s method^32,33^, as described in previous literature. Briefly, rats were anesthetized by 1% pentobarbital sodium (40 mg/kg, intraperitoneal injection). After the disappearance of corneal reflex, a 2 cm incision was made in the posterior skin at the T8-10 vertebral level. The T9 vertebral plate was removed to expose the spinal cord. Subsequently, a 3 mm diameter, 10 g weight rod was freely dropped from a height of 2.5 cm, aiming at the T9 spinal cord. Successful modeling was immediately characterized by congested and edematous spinal cord at the impact site, accompanied by hind limb extension and tail flick reflex in rats. Furthermore, upon awakening from anesthesia, the rats exhibited dragging of the hind limbs. In contrast, the rats in sham group were accepted the T9 laminectomy only without any spinal cord impact procedure. After surgery, rats continued to be bred for a week. Bladder emptying was performed twice daily by applying abdominal pressure until voluntary urination was achieved or euthanasia was carried out.

### Total RNA extraction and quantitative real-time PCR

After perfusing the rat with pre-chilled physiological saline solution through the heart, the spinal cord was harvested in an icebox. According to the protocol provided by the manufacturer, total RNA from spinal cord tissues was extracted using TRIzol (Life Technologies, USA). For every 20 mg spinal cord tissue, add 1 ml of TRIzol for RNA extraction. Complementary DNA was synthesized from total RNA with a reverse transcription kit (Thermo Fisher Scientific, USA). Real-time qPCR was executed with SYBR qPCR Master Mix (Vazyme, China). GAPDH was used as an internal reference gene, and 2^–ΔΔCt^ was used to quantify the relative expression level of genes. The primer sequences were listed in the supplementary materials (Supplementary Table 2).

### Western blot

In order to assess the expression levels of key genes, we used Western blot (WB) to validate the protein expression levels encoded by those genes. After homogenizing spinal cord tissue obtained from rats, the tissue was lysed using RIPA lysis buffer (Beyotime, China) (20 mg of spinal cord tissue mixed with 200 μl of lysis buffer and 2 μl of protease inhibitor). Total proteins were collected after centrifugation, and the protein concentration was determined using the BCA method (Bioss, China). Subsequently, 40 μg of total protein was loaded onto an SDS-PAGE gel for protein separation. Following that, the proteins were transferred to a PVDF membrane at low temperature. After blocking with 5% skim milk for 2 h, the membrane was incubated with the primary antibody overnight on a shaker at 4 °C. Following three washes with TBST, the membrane was incubated with the secondary antibody for 1 h at room temperature. Subsequently, the membrane was washed three times with TBST. Finally, protein expression levels were visualized and analyzed using enhanced chemiluminescence technology and Image J software. The GAPDH was used as an internal reference gene. The detailed information on the manufacturer and dilution ratio of the primary antibody was listed in the supplementary materials (Supplementary Table 3).

### Statistical analysis

All results are presented as the mean ± standard error of the mean (M ± SEM). Statistical analysis was performed in GraphPad Prism 7.0 (GraphPad Software Inc., San Diego, CA). Comparisons between the two groups were performed using Student’s t test or the Mann–Whitney test, as appropriate. Graphs were made using R software (Version 3.6.0). A *P* value < 0.05 was considered to be statistically significant. *p < 0.05, **p < 0.01, ***p < 0.001, and ****p < 0.0001.

### Ethics approval and consent to participate

The study protocol was approved by the Research Ethics Committee of The Second Xiangya Hospital, Central South University, Hunan, P.R. China.

## Results

### Identification of PRGs in SCI

**Figure 1:**
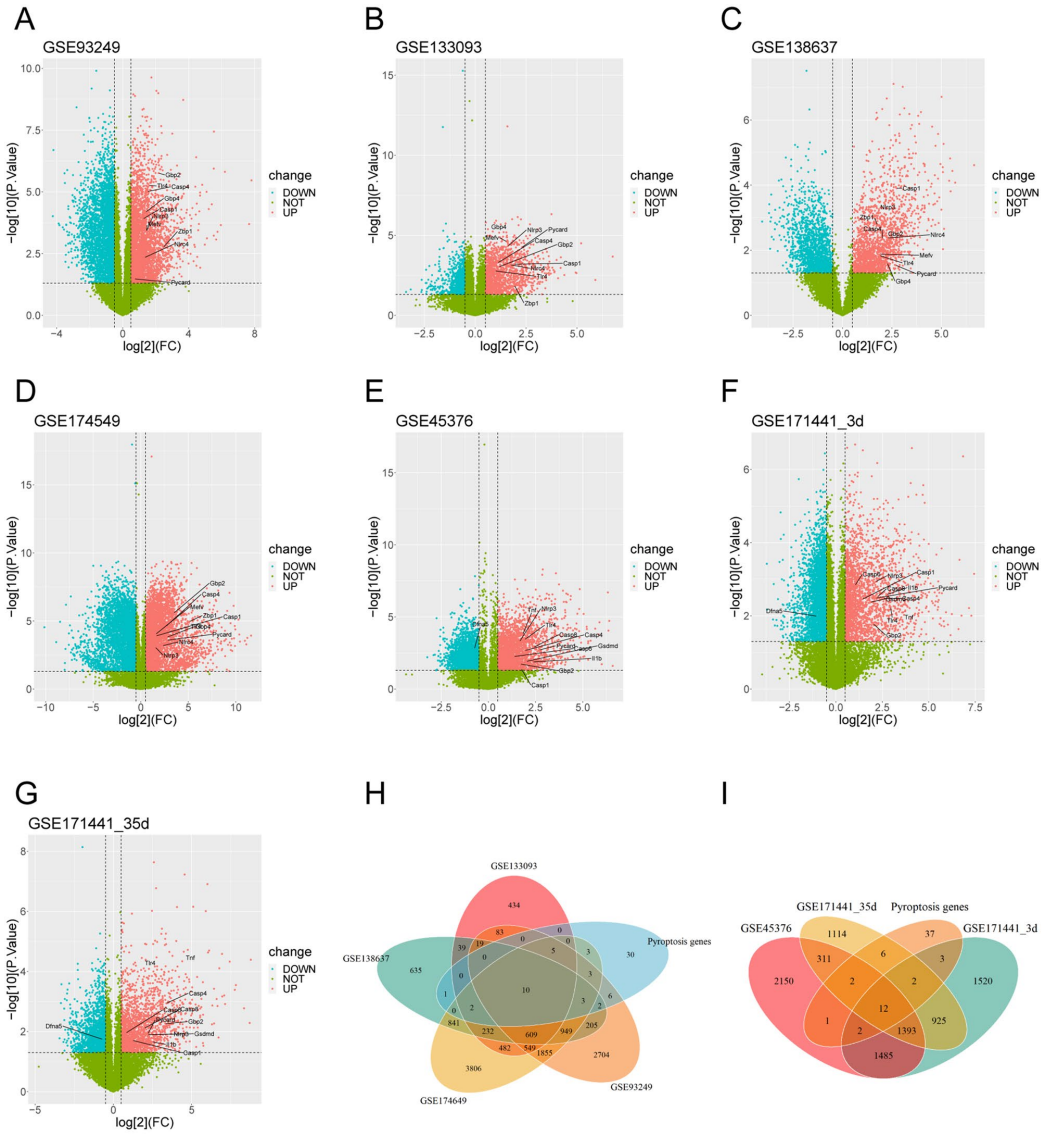
The seven volcano plots show differentially expressed genes (DEGs) between the sham group and SCI group. The criterion of |log_2_ (fold change) ≥ 0.5| and *P* value < 0.05 was used. The red, green, and blue points represent up-regulated genes, genes with no significant difference, and down-regulated genes, respectively. (**A**–**D**) are derived from rat database in the GEO database, (**E**–**G**) are from the mouse dataset. Veen diagrams separately display the intersections between DEGs and pyroptosis-related genes (PRGs) in rats and mice. There are 10 intersections genes (**H**) in rats and 12 intersections (**I**) in mice.

**Figure 2:**
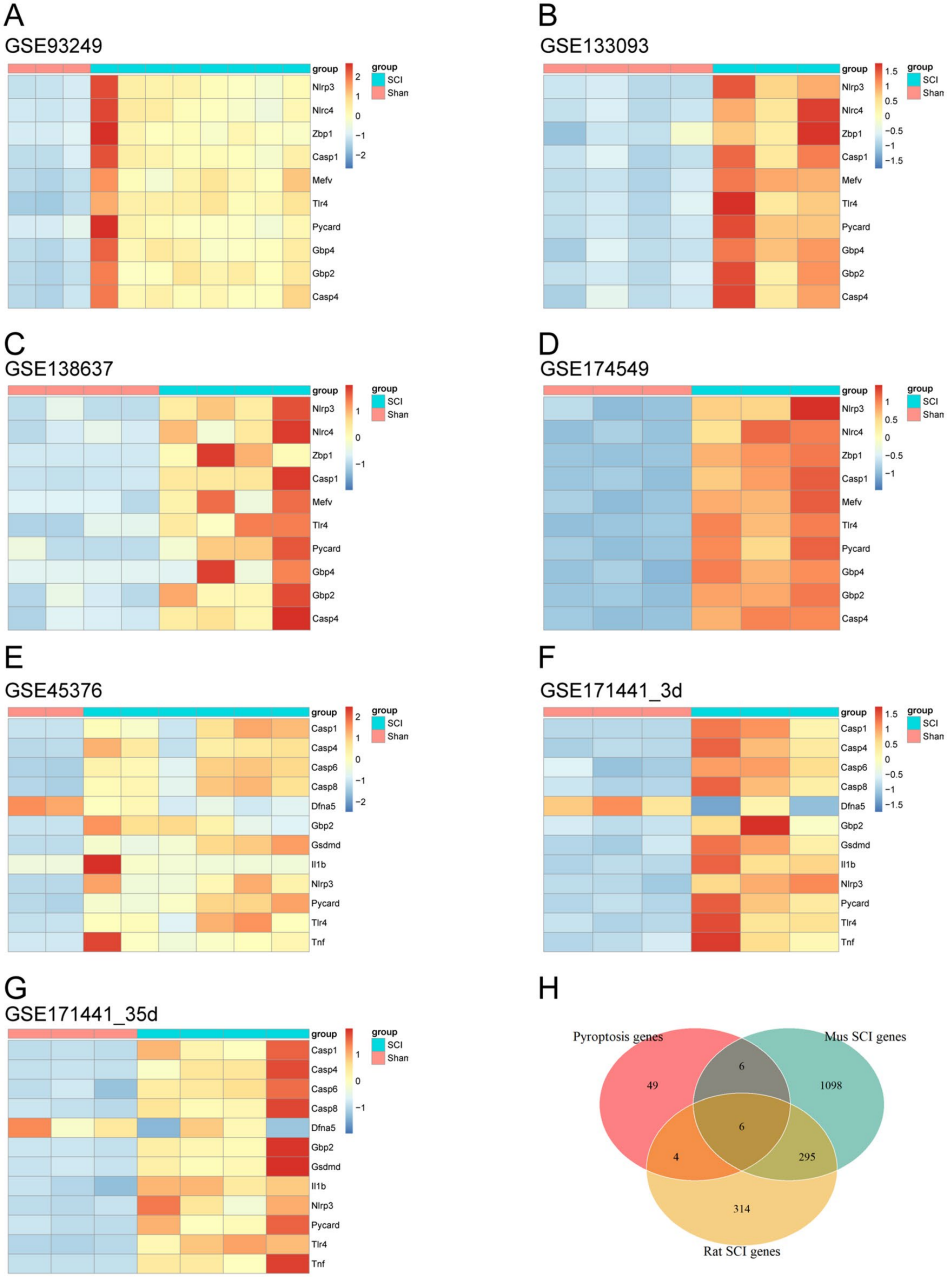
The seven heatmaps show that these pyroptosis-related DEGs exhibit distinct difference between the sham group and SCI group (**A**–**G**). The Venn diagram illustrates the intersections between DEGs in rats and mice, as well as PRGs. A total of 6 intersecting genes have been identified (**H**).

The gene expression profiles of GSE93249, GSE133093, GSE138637, GSE174549, GSE45376, GSE171441_3d and GSE171441_35d were selected in this study. These datasets contained respectively 11, 7, 8, 6, 8, 6, 7 samples. There were 7002, 2464, 3547, 9349, 5356, 5342 and 3762 DEGs after SCI respectively (*P* < 0.05 and |log FC|≥ 0.5). In the volcano plot, every plot indicated a gene and the blue plots were downregulated, the red plots were upregulated (Fig. 1A–G). We have identified a total of 65 PRGs from the existing literature, which will be used for subsequent comprehensive analysis (Supplementary Table 1). Veen analysis was performed to get the intersection of these DEGs with PRGs (Fig. 1H and I). Results showed that there were 10 intersection genes in rat datasets and 12 in mouse datasets. Then, heatmaps demonstrated that the expression difference of these genes between Sham group and SCI group was very pronounced (Fig. 2A–G). Finally, Venn diagram showed 6 common pyroptosis-related DEGs between rat and mouse (Fig. 2H).

### GO (gene ontology) and KEGG enrichment analysis of pyroptosis-related DEGs

The differentially expressed PRGs were submitted to Metascape for KEGG enrichment and GO enrichment analysis involving Biologocal Process (BP), cellular composition (CC) and molecular function (MF) and visualized by using R software.

**Figure 3:**
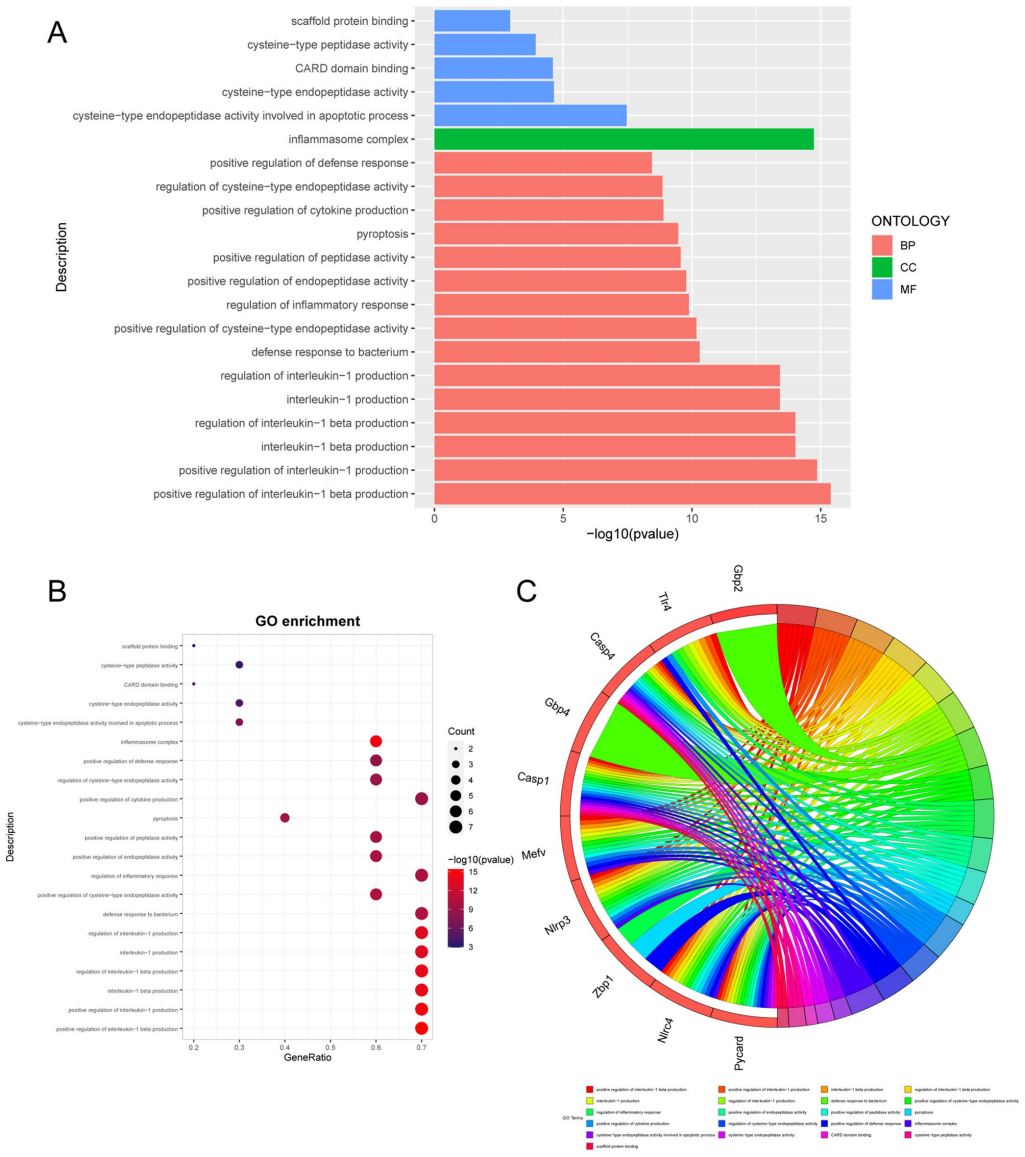
GO annotation of 10 pyroptosis-related DEGs of rat datasets following SCI. The bar charts (**A**) of the top 15 GO terms were drawn on the basis of *P*-value and the percentage of genes; terms with *P*-value < 0.01 are statistically significant. The bubble plot visually presents the GO terms in the enrichment analysis, with the size and color coding indicating the degree of enrichment and significance level (**B**). The chord plot shows the PRGs enriched in different GO terms (**C**).

**Figure 4:**
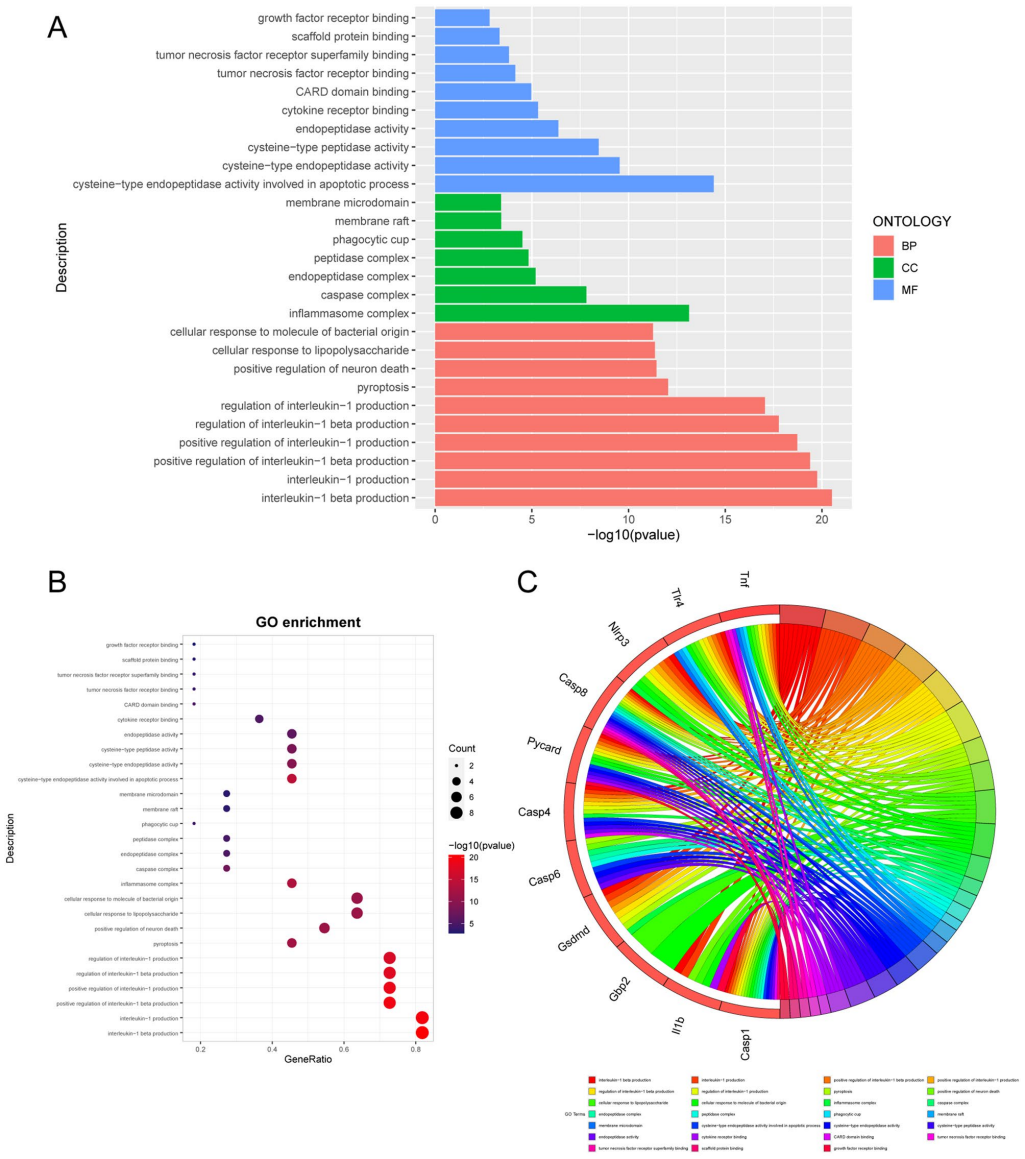
GO annotation of 12 pyroptosis-related DEGs of mouse datasets following SCI. The bar charts (**A**) of the top 15 GO terms were drawn on the basis of *P*-value and the percentage of genes; terms with *P*-value < 0.01 are statistically significant. The bubble plot visually presents the GO terms in the enrichment analysis, with the size and color coding indicating the degree of enrichment and significance level (**B**). The chord plot shows the PRGs enriched in different GO terms (**C**).

**Figure 5:**
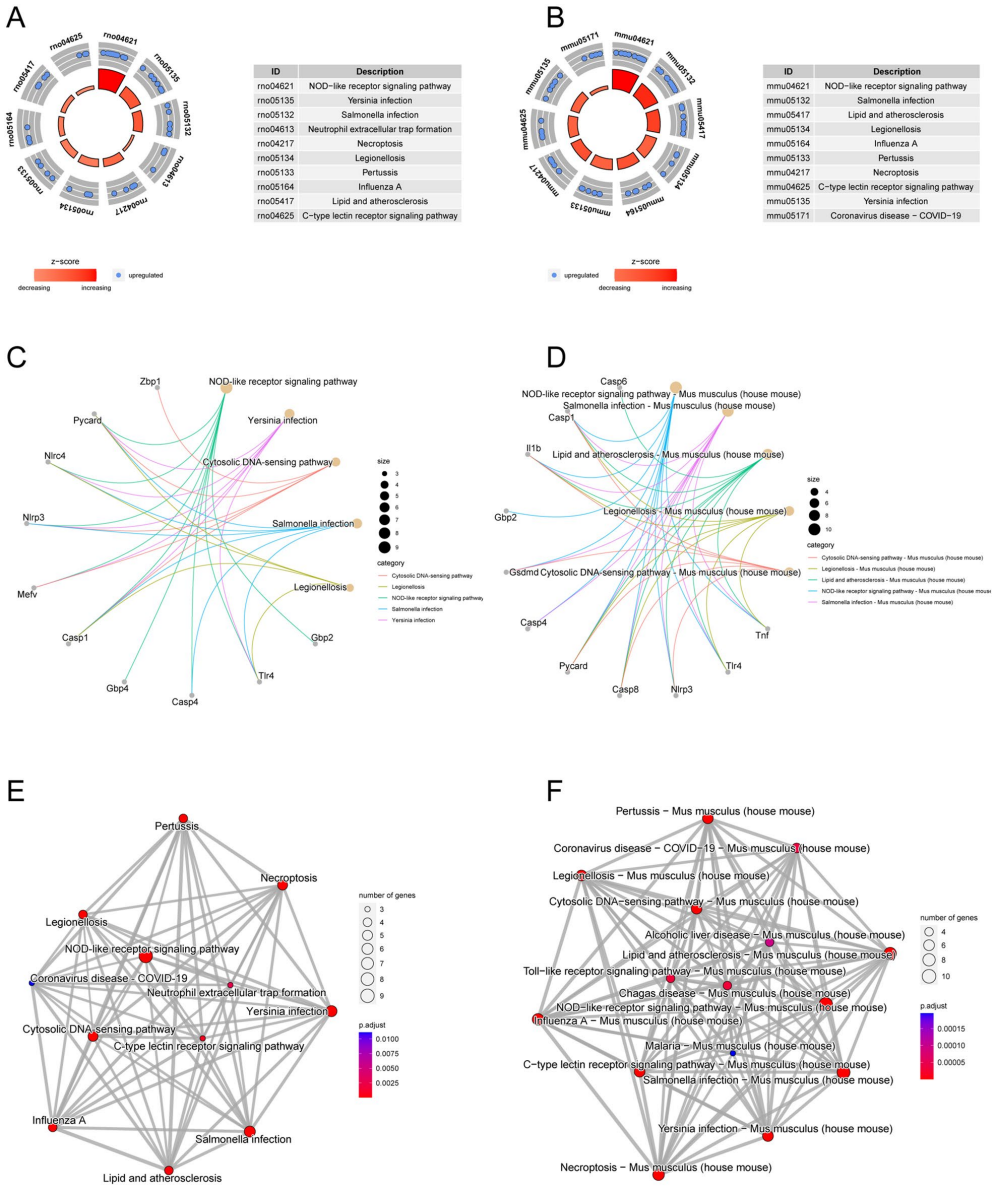
The top 10 KEGG pathways for DEGs in rats and mice datasets. The chord diagram displays the association between the PRGs and KEGG pathway, while also indicating the number of enriched genes ((**A**) from rat, (**B**) from mouse). (**C**) and (**D**) visualizes the association network between PRGs and their corresponding pathways in rat and mouse respectively. (**E**) and (**F**) respectively demonstrate the interconnections between pathways in rats and mice.

In the rat datasets, the top 15 eligible terms for GO analysis of pyroptosis-related DEGs were shown in Fig. 3A, and the number of enriched genes and adjusted *P*-value were shown in Fig. 3B. From the figures, it is evident that the pyroptosis-related DEGs were primarily enriched in interleukin-1 production and regulation, inflammatory responses, pyroptosis, inflammasome complex and cysteine-type endopeptidase actively involved in apoptotic process. The relationship of genes and GO terms was shown by Chord plot (Fig. 3C), which allows for a more intuitive visualization of the relationship between genes and pathways. In the KEGG enrichment analysis the pyroptosis-related DEGs were mainly enriched in the following top 10 pathways: NOD-like receptor signaling pathway, Yersinia infection, Salmonella infection, Neutrophil extracellular trap formation, Necroptosis, Legionellosis, Influenza A, Lipid and atherosclerosis and C-type lectin receptor signaling pathway (Fig. 5A). Figure 5C and E, respectively, demonstrate the relationship between genes and pathways, as well as the relationship between pathways themselves. While, in the mouse datasets, similar results were obtained in terms of GO (Fig. 4A–C) and KEGG (Fig. 5B,D and F) enrichment analyses, with only slight variations in the ordering of presentation. From these results, it can be seen that in both rat and mouse SCI, PRGs primarily participate in inflammatory processes, programmed cell death, and defense-related physiological and pathological processes.

### Correlation analysis based on pyroptosis-related DEGs and PPI network

**Figure 6:**
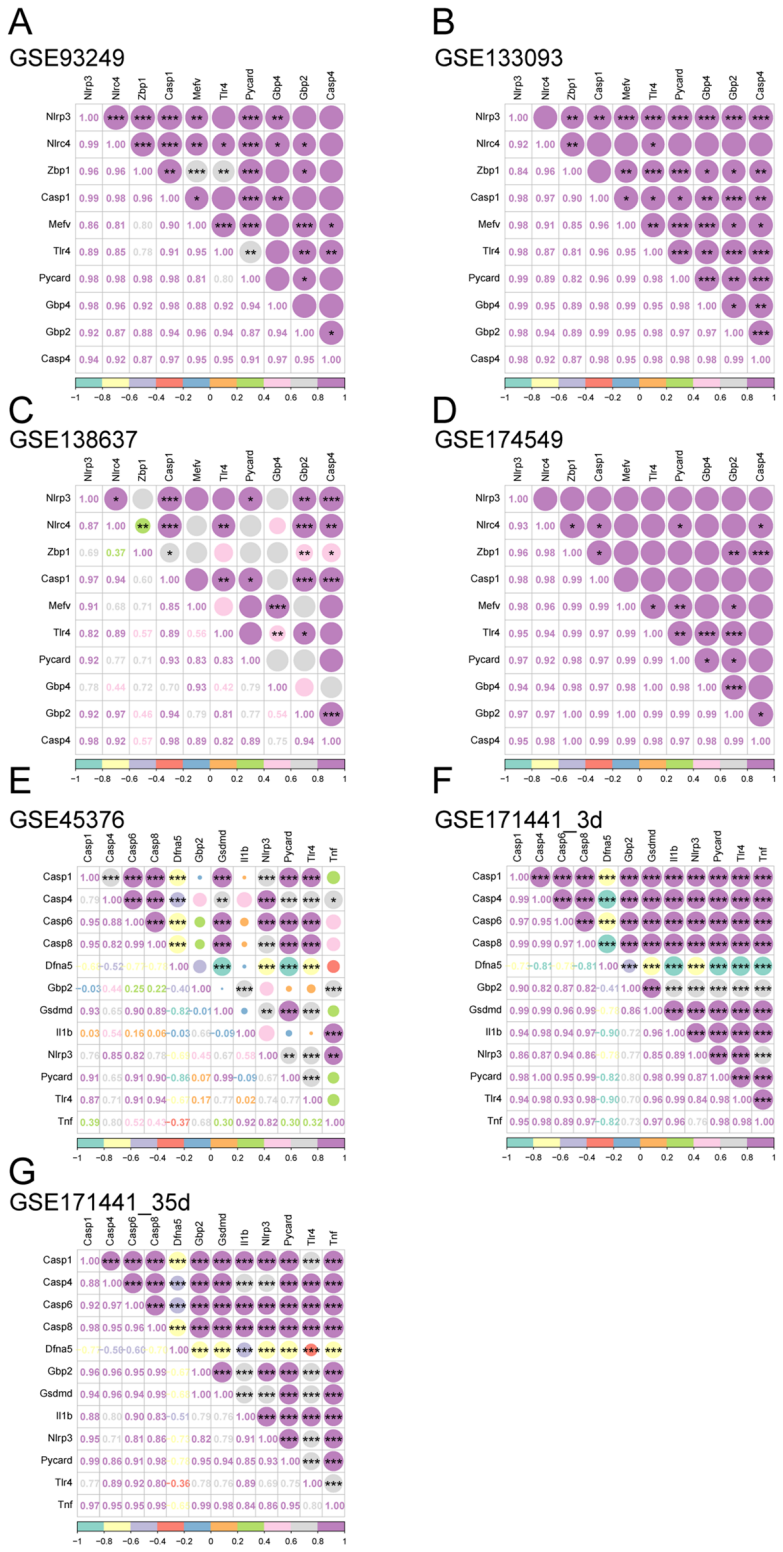
The Spearman correlation analysis of the pyroptosis-related DEGs. (**A**–**D**) are derived from rat database in the GEO database, (**E**–**G**) are from the mouse dataset. *P < 0.05, **P < 0.01, ***P < 0.001.

**Figure 7:**
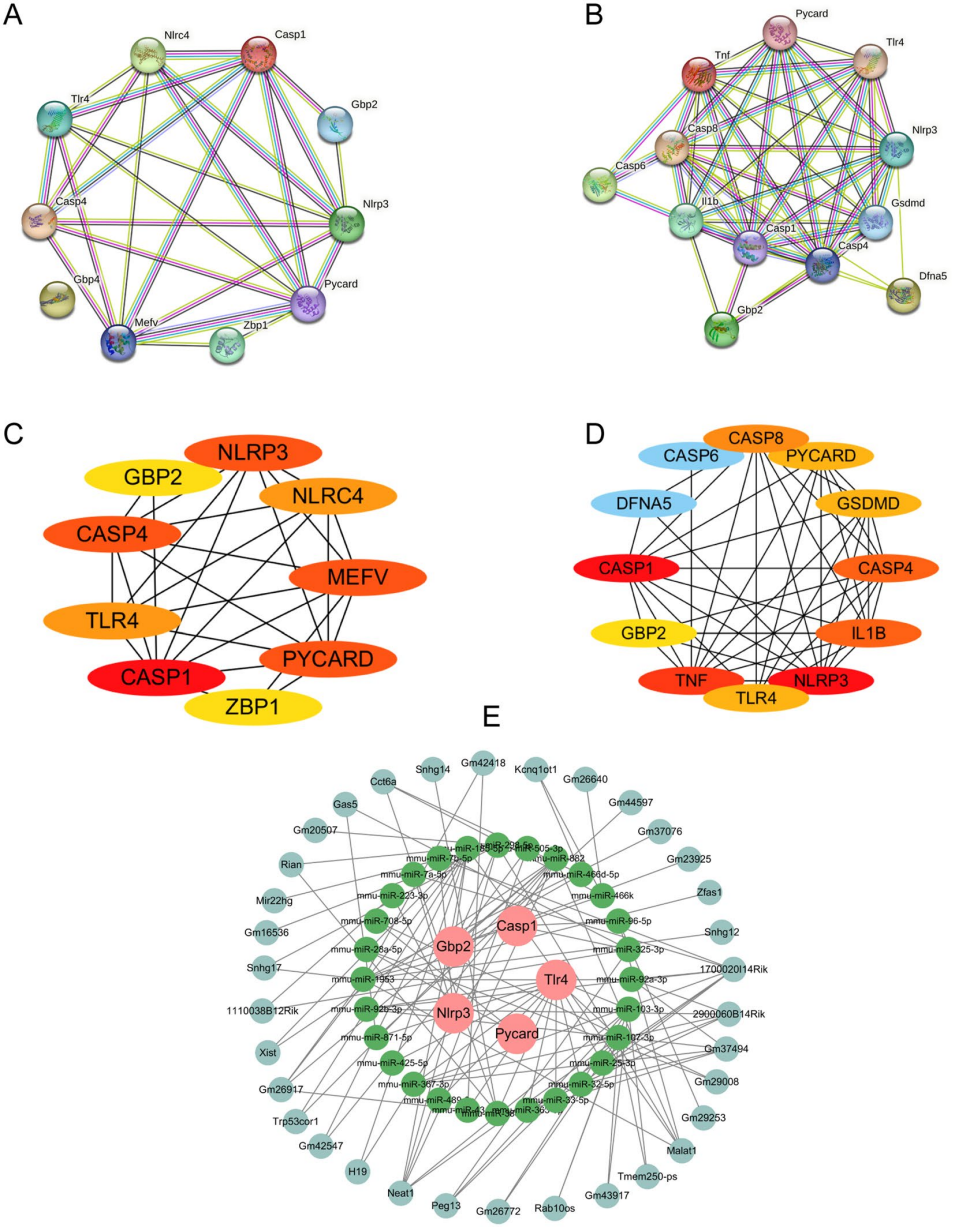
PPI network of pyroptosis-related DEGs identified were obtained from the STRING database (**A**,**B**). Hub genes identified by Cytoscape software, the higher the ranking, the redder the color (**C** and **D**) ((**A**) and (**C**) is derived from rat database in the GEO database, (**B**) and (**D**) is from the mouse dataset). Pyroptosis-related mRNA-miRNA-lncRNA regulatory networks in SCI (**E**). The pink circles represent PRGs, the green circles represent miRNAs, and the pale green circles represent lncRNAs. *lncRNA* long non-coding RNA, *miRNA* microRNA, *SCI* spinal cord injury.

The correlation analysis among these pyroptosis-related DEGs expression was shown in Fig. 6. The results demonstrate significant correlations between genes, particularly Casp1 and NRPL3, which exhibit significant and meaningful correlations with other genes. This suggests potential regulatory or synergistic interactions among these PRGs. To explore the interactions between the proteins expressed by the pyroptosis-related DEGs, we constructed a PPI network using the STRING database. Cytoscape (version 3.7.2) was used for visualization of PPI network. The PPI network of rat pyroptosis-related DEGs contained 7 nodes and 27 linkages (Fig. 7A), while 11 nodes and 46 linkages contained in mouse (Fig. 7B). The PPI data from both rats and mouses were separately imported into Cytoscape software to identify hub genes (Fig. 7C and Fig. 7D). The intersection of the top 5 hub genes was selected for subsequent validation experiments. We identified a total of three common hub genes, namely Casp1, Casp4 and Nlrp3 for subsequent study.

### Construction of the lncRNA-miRNA-mRNA regulatory network related to pyroptosis

We performed miRNA and lncRNA predictions for the 6 intersecting genes (Casp1, Gbp2, Nlrp3, Pycard, Tlr4 and Casp4) obtained from Venn diagram analysis using the starBase (Version 2.0) website. However, no miRNA or lncRNA predictions were obtained for Casp4 gene. We predicted a total of 28 miRNAs and 34 lncRNAs. These data were imported into Cytoscape software to generate the ceRNA regulatory network (Fig. 7E). This regulatory network elucidated the potential regulatory mechanisms of pyroptosis in SCI.

### Validation of hub genes for pyroptosis in SCI by qPCR and WB

**Figure 8:**
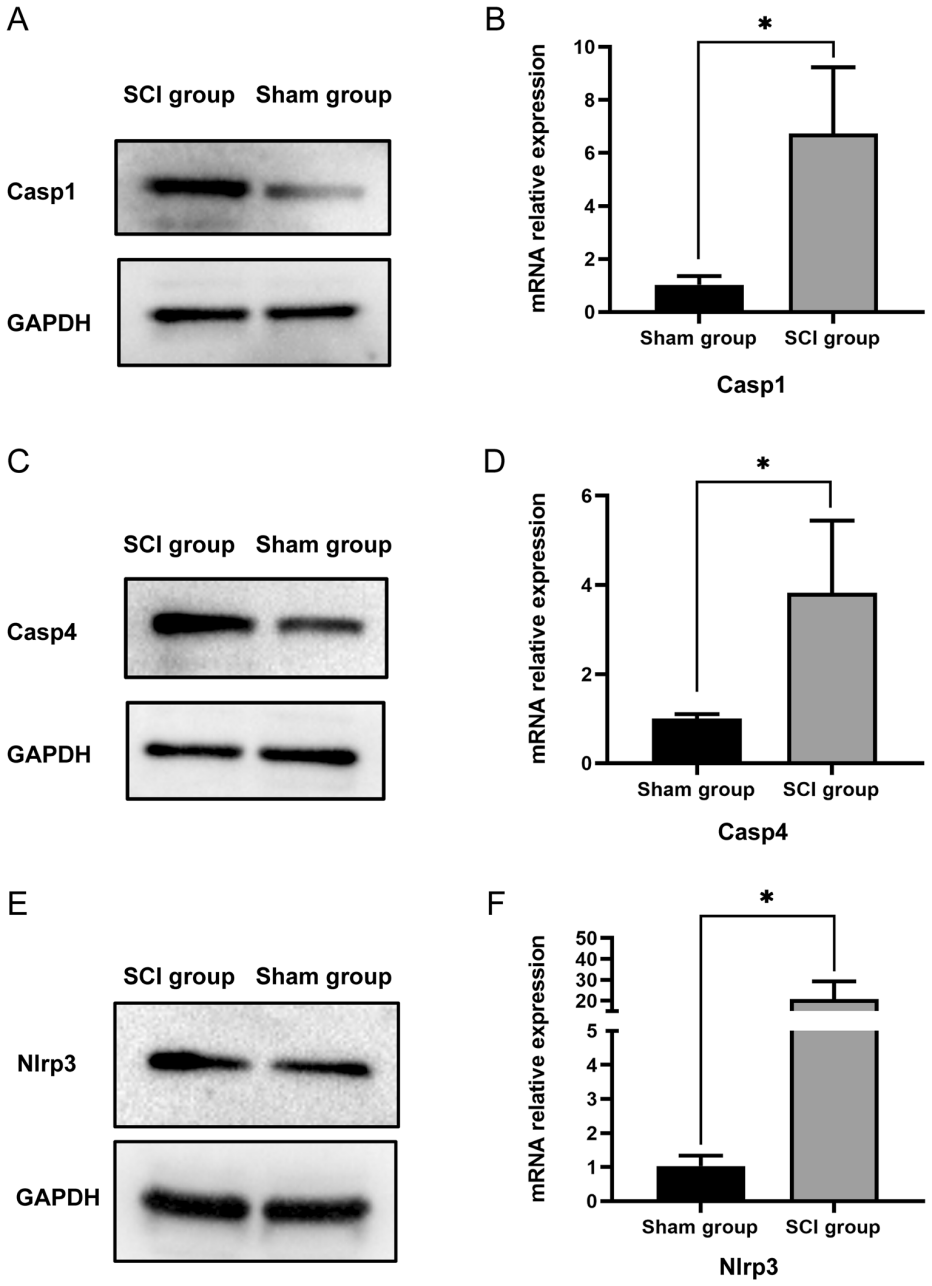
WB (**A**, **C** and **E**) and qPCR (**B**, **D** and **F**) were used to validate the expression of PRGs in rat spinal cord tissue. SCI (spinal cord injury). (The three target gene bands originated from different gels. The gels/blots were cropped, and the original blots are presented in Supplementary Figures. S2–S4) *P < 0.05, **P < 0.01, ***P < 0.001.

The three hub genes (Casp1, Casp4 and Nlrp3) were validated in tissue samples. As shown in Fig. 8, compared to the sham surgery group, the three hub genes in the SCI group exhibited high expression at both the RNA and protein levels, and the differences were statistically significant. These findings are consistent with the results of previous data analysis, indicating an upregulation of PRGs in spinal cord injury and suggesting that inhibiting the expression of PRGs may contribute to the recovery of spinal cord function.

## Discussion

In this study, we performed bioinformatics analysis of mRNA sequencing data from rat and mouse models of SCI, which were obtained from the GEO database. We integrated these data with PRGs, which have been the focus of recent research. Our analysis revealed differential expression of PRGs in both rat and mouse SCI models, with 10 and 12 DEGs, respectively. The majority of these genes exhibited high expression levels both rat and mouse models of SCI, except for one gene (Dfna5) showing low expression in the mouse injury model. Six common differentially expressed genes were identified in the intersection of rat and mouse PRGs. Enrichment analysis of these DEGs indicated that GO analysis was mainly focused on inflammation-related factors, while KEGG analysis showed that the most genes were enriched on the NOD-like receptor signaling pathway. Our study represents the integration of analysis of SCI and PRGs, and our results suggest that pyroptosis may be one of the specific mechanisms underlying secondary spinal cord injury. Therefore, inhibiting the high expression of PRGs may be a novel therapeutic target for SCI treatment, and our findings have significant implications in this area.

The mechanism of secondary spinal cord injury is highly complex. Currently, there is a considerable amount of evidence indicating the involvement of programmed cell death in the occurrence and progression of secondary spinal cord injury^34^. These programmed cell death processes include autophagy, apoptosis, ferroptosis, necroptosis, parthanatos, and pyroptosis^34^. Among these processes, autophagy and apoptosis have been extensively studied and researched in SCI, while pyroptosis, as a relatively recent research focus, has also gained increasing attention. With the elucidation of the mechanisms of pyroptosis in SCI becoming increasingly clear, it is now known that changes in PRGs may have various effects on neuronal cells. Moreover, pyroptosis does not occur as an independent event but often interacts with other forms of programmed cell death, forming a complex network of interactions. Following SCI, a large amount of reactive oxygen species accumulates in cells, which can induce the activation of the NLRP3 inflammasome, subsequently activating Caspase-1. Mature Caspase-1, on one hand, triggers cell pyroptosis by cleaving Gasdermin proteins, and on the other hand, it can weaken the TRIF/Toll-like receptor 4 signaling pathway, inducing cell autophagy^35,36^. Cell autophagy, in turn, can eliminate damaged organelles (such as mitochondria) and inflammatory factors, thereby negatively regulating NLRP3 and inhibiting cell pyroptosis^36^. Xu et al.^37^ found that Toll-like receptor 4 (TLR4) plays an important role in SCI, and inhibiting the expression of TLR4 can alleviate neuron pyroptosis. Additionally, they discovered that the expression levels of NLRP3/GSDMD proteins in the peripheral blood of SCI patients were higher compared to normal patients, and the expression levels were positively correlated with the severity of the injury, suggesting that pyroptosis may aggravate SCI. In this study, we integrated and analyzed multiple datasets from rats and mice, and found differential expression of PRGs in SCI of both species. Furthermore, high expression was observed at days 3 and 35 post-injury in the datasets (GSE171441), indicating that pyroptosis may play an important role in the acute and subacute stages of the disease course. In the correlation analysis (Fig. 6), it can be observed that Caspase-1 exhibits significant correlations with Nlrp3, Pycard, and others, suggesting a possible synergistic or regulatory relationship between them. In conclusion, the role of pyroptosis and its molecular regulation in SCI are highly complex, requiring further in-depth and extensive research.

Pyroptosis is a current research hotspot. It is worth noting that there have been few reports on the study of PRGs in SCI using bioinformatics analysis. In this study, we identified differentially expressed PRGs in SCI through transcriptomic data analysis and joint analysis of multiple datasets. Among them, 10 genes were identified in rats and 12 genes in mice, with an intersection of 6 genes (Fig. S1). Subsequently, GO and KEGG enrich analysis were performed on the PRGs in rats and mice separately to understand their functions and regulatory mechanisms in biological processes. GO analysis indicated that these genes are mainly enriched in pathways related to inflammation, such as the inflammasome complex, interleukin-1 beta production, interleukin-1 production, and positive regulation of interleukin-1 beta production. KEGG analysis suggested that these genes are mainly enriched in the NOD-like receptor signaling pathway, necroptosis, and infection, both in rats and mice. These results indicate that pyroptosis does occur in SCI and may play an important role.

The role of non-coding RNAs in SCI is increasingly being studied, especially miRNAs and lncRNAs. A substantial body of research has demonstrated their widespread involvement in the pathological and physiological processes of SCI, making them potential new targets for therapeutic interventions. miRNAs play an important regulatory role in neuronal apoptosis, the release of inflammatory factors, and oxidative stress following SCI. Liu et al.^38^ found that the expression level of miRNA-233 significantly increased in a rat SCI model. Intrathecal injection of the miRNA-233 inhibitor, antagomir-223, significantly reduced the expression of apoptosis-related proteins (Bax and cleaved caspase-3), leading to a significant improvement in hindlimb motor function recovery after SCI. Furthermore, studies have shown that miRNAs such as miR-34a, miR-92b-3p, miR-29a, miR-181d-5p, miRNA-99b-5p, miRNA-125b, miRNA-21, miRNA-212-3p, and miRNA-26a are involved in the regulation of neuronal apoptosis following SCI. Fei et al.^39^ demonstrated that overexpression of miRNA-82 can attenuate the release of inflammatory cytokines (TNF-a, IL-6, IL-1β, etc.) by blocking the IKKβ/NF-kB pathway, thereby improving spinal cord edema and functional recovery in mice after SCI. Wang et al.^40^ found that miRNA-99a can alleviate oxidative stress and promote functional recovery in rats after SCI by inhibiting the expression of NOX4. The miRNAs exert both positive and negative regulation in SCI, and their mechanisms are highly complex, requiring further in-depth research. It has been discovered that lncRNAs, similar to miRNAs, play an important role in the regulation following SCI and have close interactions with miRNAs^41^. LncRNAs XIST, BDNF-AS, and MALAT1 are involved in the regulation of neuronal apoptosis after spinal cord injury^42,43^. Among them, lncRNA XIST can induce neuronal apoptosis by inhibiting the expression of miRNA-7a^42^. Additionally, similar to miRNAs, lncRNAs can also regulate inflammatory factors and oxidative stress after SCI. In this study, we predicted and identified 28 miRNA and 34 lncRNA through 5 key PRGs. We constructed an mRNA-miRNA-lncRNA regulatory network, which revealed close relationships among them. This regulatory network may represent potential regulatory mechanisms underlying SCI and deserves further investigation.

The involvement of pyroptosis-related signaling pathways in SCI is currently extensive, and the effects of various signals may differ. The PI3K/AKT signaling pathway^37,44^ and PI3K-AKT-Foxo1 have been found by Hu^44^ and Xu et al.^45^, to demonstrate that Taxifolin and CD73 (an immunosuppressive molecule) can alleviate inflammation and microglia-mediated pyroptosis after SCI through the PI3K/AKT signaling pathway, thereby improving spinal cord function. The STING signaling pathway^46^, discovered by scholars has been found to amplify inflammatory responses and induce pyroptosis^46,47^. Additionally, Liu and others have found that inhibiting the MAPKs-NF-κB signaling pathway can alleviate neuroinflammation and pyroptosis mediated by microglia after SCI^48,49^. Of course, many other signaling pathways have been discovered in SCI, such as the JAK2/STAT1 signaling pathway^50^, AMPK/NLRP3 signaling pathway^51^, AMPK-mTOR-TFEB signaling pathway^52^, and AIM2/ASC/Caspase-1 signaling pathway^53^. In our research, the PRGs enriched the most in the KEGG enrichment analysis were associated with the NOD-like receptor signaling pathway, both in rats and mice data. The NOD-like receptor signaling pathway, within the NOD-like receptor protein family, plays a crucial role in pyroptosis by mediating the assembly of inflammasomes, the activation of Caspase-1, the release of pro-inflammatory cytokines, and inducing cell membrane rupture through GSDMD cleavage. Among the NOD-like receptor protein family, the most relevant inflammasomes to the central nervous system are NLRP1 and NLRP3. Our analyzed data showed significant differences in NLRP3 expression, which closed correlated with SCI.

In conclusion, the specific molecular regulatory mechanisms of pyroptosis in SCI are complex and not yet fully understood. Further research involving extensive molecular exploration and experimental validation is still needed to investigate its role in improving the function of the injured spinal cord.

## Conclusions

Pyroptosis is involved in secondary SCI and likely plays a significant role in this process. The majority of PRGs exhibit elevated expression levels in SCI. Inhibiting the expression of PRGs may contribute to the recovery of SCI. The specific regulatory mechanisms of pyroptosis in SCI are complex and require further in-depth research.

### Supplementary Information

Supplementary Information.

## Data Availability

All data relevant to the study are included in the article or uploaded as supplementary information.
