# Supplementary material for: Identification and bioinformatics analysis of genes associated with pyroptosis in spinal cord injury of rat and mouse
Source: Sci Rep. 2024 Jun 18;14:14023. doi: 10.1038/s41598-024-64843-6 (PMC11189416; doi:10.1038/s41598-024-64843-6)

Supplementary materials

Supplementary table 1 Compilation of pyroptosis-related genes. (Among these genes, there are three pairs of genes with different names, marked as “[#]”, “[¥]”, “[*]” respectively, but it does not affect subsequent analysis and processing.)

Gene symbol	Gene name
Gsdma	gasdermin A
Gsdmb	gasdermin B
Gsdmc	gasdermin C
Gsdmd	gasdermin D
[#]Gsdme	gasdermin E
Il-1b	interleukin 1 beta
Il18	interleukin 18
Casp1	caspase-1
Casp3	caspase-3
Casp4	caspase-4
Casp5	caspase-5
Casp6	caspase-6
Casp8	caspase-8
Gzma	granzyme A
Gzmb	granzyme B
Hmgb1	high mobility group box 1
Gbp1	guanylate binding protein 1
Gbp2	guanylate binding protein 2
Gbp3	guanylate binding protein 3
Gbp4	guanylate binding protein 4
Gbp5	guanylate binding protein 5
Ddx3x	DEAD-box helicase 3 X-linked
Naip	NLR family apoptosis inhibitory protein
Serpinb1	serpin family B member 1
C-Flip	cellular FADD-like interleukin-1b converting enzyme inhibitory protein
Tnfrsf21	TNF receptor superfamily member 21
Irf2	interferon regulatory factor 2
Zbp1	Z-DNA binding protein 1
Prf1	perforin 1
Dhx9	guanylate binding protein 5
[*]Dfnb59	DFNB59 (pejvakin)
Ctsg	cathepsin G
Apip	APAF1 interacting protein
Aim2	absent in melanoma 2

Nlrc4	NLR family CARD domain containing 4
Nlrp1	NLR family pyrin domain containing 1
Nlrp2	NLR family pyrin domain containing 2
Nlrp3	NLR family pyrin domain containing 3
Nlrp6	NLR family pyrin domain containing 6
Nlrp7	NLR family pyrin domain containing 7
Nlrp9	NLR family pyrin domain containing 9
Nlrp12	NLR family pyrin domain containing 12
Nlrx1	NLR family member X1
Nod2	nucleotide binding oligomerization domain containing 2
Tlr4	toll like receptor 4
$Mefv	MEFV innate immuity regulator (Pyrin)
Pydc1	pyrin domain containing 1
Pycard	PYD and CARD domain containing
Card8	caspase recruitment domain family member 8
Card16	caspase recruitment domain family member 16
Card18	caspase recruitment domain family member 18
#Dfna5	Deafness, autosomal dominant 5
*Pjvk	Pleckstrin homology domain-containing, family V, member 1
Casp9	caspase-9
Elane	Elastase, neutrophil expressed
Gpx4	Glutathione peroxidase 4
$Pyrin	also known as MEFV (Mediterranean fever gene
Il6	Interleukin 6
Nod1	Nucleotide-binding oligomerization domain-containing protein 1
Plcg1	Phospholipase C gamma 1
Prkaca	Protein kinase cAMP-activated catalytic subunit alpha
Pycard	PYD and CARD domain-containing
Scaf11	Scaffold protein 11
Tirap	Toll-interleukin 1 receptor domain-containing adaptor protein
Tnf	Tumor Necrosis Factor

Supplementary table 2 Lists of primer sequences used for quantitative real-time PCR

Genes	Sequences
Casp1	Fprimer: GAAGATGATGGCATTAAGAAGG
	Rprimer: CCAGGACACATTATCTGGTG
Casp4	Fprimer: CTACTCTACAACACCACACCA
	Rprimer: AAGCAGGAAATGAGTTTGGAG
Nlrp3	Fprimer: AGACATGGGACTCAAGCTC
	Rprimer: CTGCAGTTGTCTAACTCCAG
Gapdh	Fprimer: ATTCCATCCCAGACCCCATAAC
	Rprimer: GCAGCGAACTTTATTGATGGTAT

Supplementary table 3 List of antibodies

Antibody name	Cat number	Company	WB dilution
Casp1	AF1681	Beyotime	1:1000
Casp4	67398-1-lg	Proteintech	1:2000
Nlrp3	68102-1-lg	Proteintech	1:2000
Gapdh	60004-1-Ig	Proteintech	1:20000

Supplementary Figure S1

The intersection of differentially expressed genes in rats, differentially expressed genes in mice, and pyroptosis-related genes resulted in six overlapping genes.

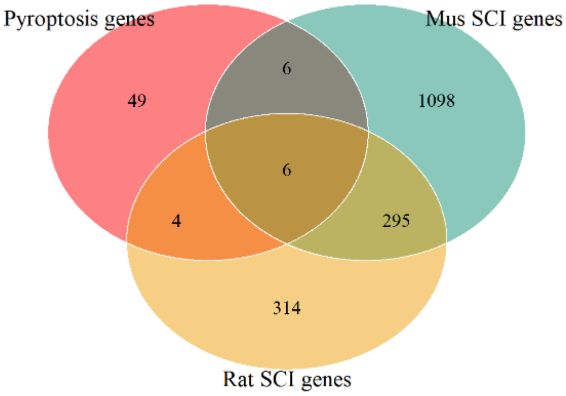

The following figure displays differentially expressed genes in rats (left) and differentially expressed genes in mice (right).

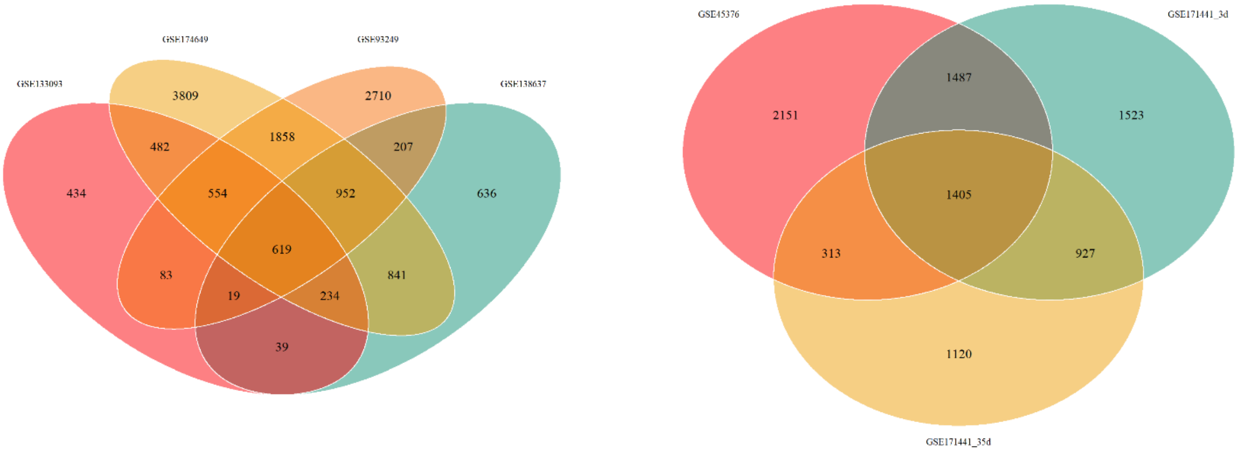

Figure S2

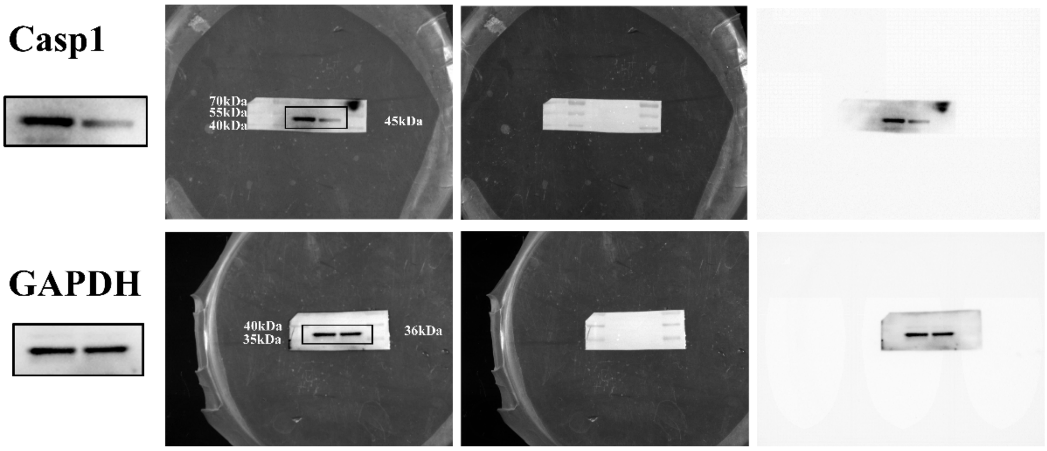

Figure S3

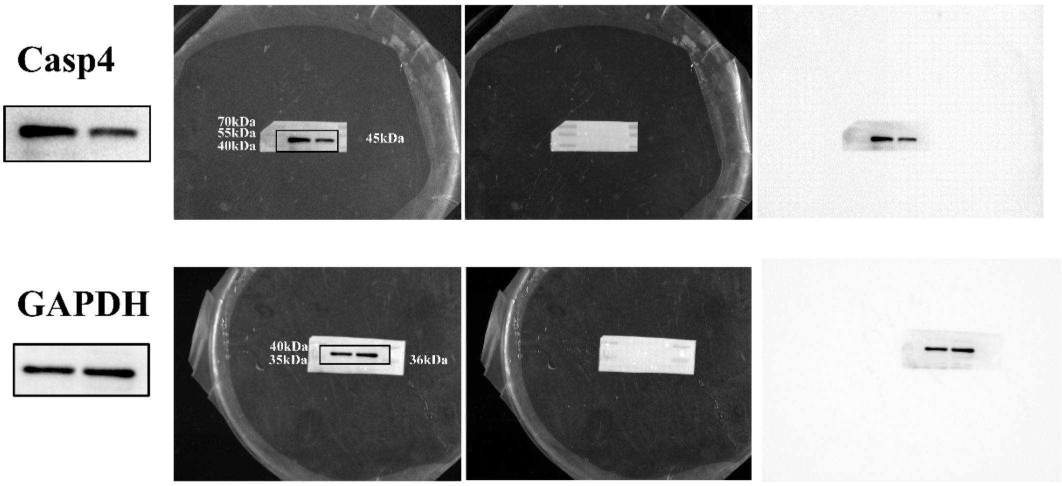

Figure S4

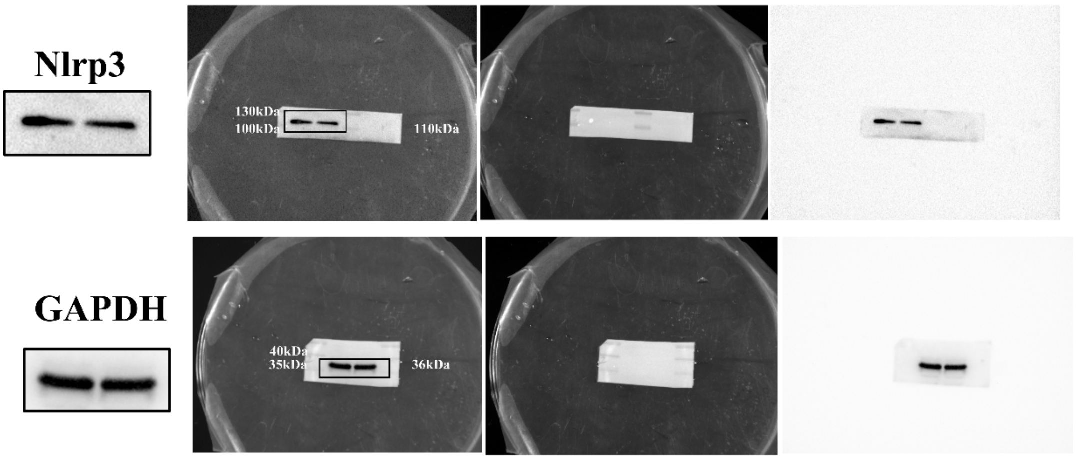

Supplement: Supplementary file 1 — Supplementary Information. [file 41598_2024_64843_MOESM1_ESM.pdf]
